# Developing and diagnosing climate change indicators of regional aerosol optical properties

**DOI:** 10.1038/s41598-017-18402-x

**Published:** 2017-12-22

**Authors:** Ryan C. Sullivan, Robert C. Levy, Arlindo M. da Silva, Sara C. Pryor

**Affiliations:** 1 Department of Earth and Atmospheric Sciences, Cornell University, Ithaca, NY USA; 2 NASA Goddard Space Flight Center, Greenbelt, MD USA; 3 Pervasive Technology Institute, Indiana University, Bloomington, IN USA; 4 Environmental Science Division, Argonne National Laboratory, Argonne, IL USA

## Abstract

Given the importance of aerosol particles to radiative transfer via aerosol-radiation interactions, a methodology for tracking and diagnosing causes of temporal changes in regional-scale aerosol populations is illustrated. The aerosol optical properties tracked include estimates of total columnar burden (aerosol optical depth, AOD), dominant size mode (Ångström exponent, AE), and relative magnitude of radiation scattering versus absorption (single scattering albedo, SSA), along with metrics of the structure of the spatial field of these properties. Over well-defined regions of North America, there are generally negative temporal trends in mean and extreme AOD, and SSA. These are consistent with lower aerosol burdens and transition towards a relatively absorbing aerosol, driven primarily by declining sulfur dioxide emissions. Conversely, more remote regions are characterized by increasing mean and extreme AOD that is attributed to increased local wildfire emissions and long-range (transcontinental) transport. Regional and national reductions in anthropogenic emissions of aerosol precursors are leading to declining spatial autocorrelation in the aerosol fields and increased importance of local anthropogenic emissions in dictating aerosol burdens. However, synoptic types associated with high aerosol burdens are intensifying (becoming more warm and humid), and thus changes in synoptic meteorology may be offsetting aerosol burden reductions associated with emissions legislation.

## Introduction

Atmospheric aerosol particles (aerosols) impact biogeochemical cycles, human health, and global and regional climate by scattering and absorbing radiation, acting as cloud condensation nuclei or ice nucleating particles and altering cloud lifetimes and albedo, and changing the atmospheric thermal structure and thus atmospheric stability (ref.^1^ and references therein). According to some estimates aerosol particles may have offset 0.9 Wm^−2^ (– 0.95 to + 0.05 Wm^−2^ and −1.2 to 0.0 Wm^−2^ for aerosol-radiation (direct) and aerosol-cloud (indirect) interactions, respectively) of the historical globally-averaged warming due to increased greenhouse gas concentrations (2.26 to 3.40 Wm^−2^)^2^. They have also been implicated as a major source of regional and sub-regional variations in trends in near-surface temperature (e.g. in the ‘warming hole’ of the central Great Plains)^3–7^.

Aerosol radiative forcing and climate impact are a function of the aerosol number concentration, size distribution, and chemical composition, and remain a major source of uncertainty in quantifying anthropogenic forcing of Earth’s climate^2^. In contrast to well-mixed greenhouse gases, as with other short-lived climate forcers, aerosols exhibit much higher spatiotemporal variability. Local primary aerosol and precursor gas emissions have a major impact on regional aerosol populations and thus climate impacts. Hence, quantifying the radiative forcing is challenging and subject to large uncertainties. For example, during 1980–2009, the global mean annual aerosol optical depth (AOD), a measure of the extinction of insolation by atmospheric aerosols and thus the reduction of radiation that reaches Earth’s surface, was unchanged (i.e. remained within ±0.01 of an estimated global average of ~ 0.15)^8^. However, mean annual AOD decreased by up to 27% over parts of the U.S. and Europe due in part to regulation of precursor and primary aerosol emissions, while mean annual AOD increased by up to 22% over countries undergoing large economic development^8–10^. Following emission reductions associated with air quality legislation (e.g., U.S. Clean Air Act)^11^, near-surface fine aerosol concentrations (PM_2.5_, i.e. the mass concentration of aerosols with diameters less than 2.5 μm) decreased by 40% across the continental U.S. during this period^8^. This is consistent with a 38% decrease in modeled AOD from 1980–2006 (ref.^12^), and ~3% yr^−1^ decrease in summer AOD over the eastern U.S. from 2001–2013 retrieved using satellite-based remote sensing (the Multi-angle Imaging SpectroRadiometer (MISR))^13^.

**Figure 1:**
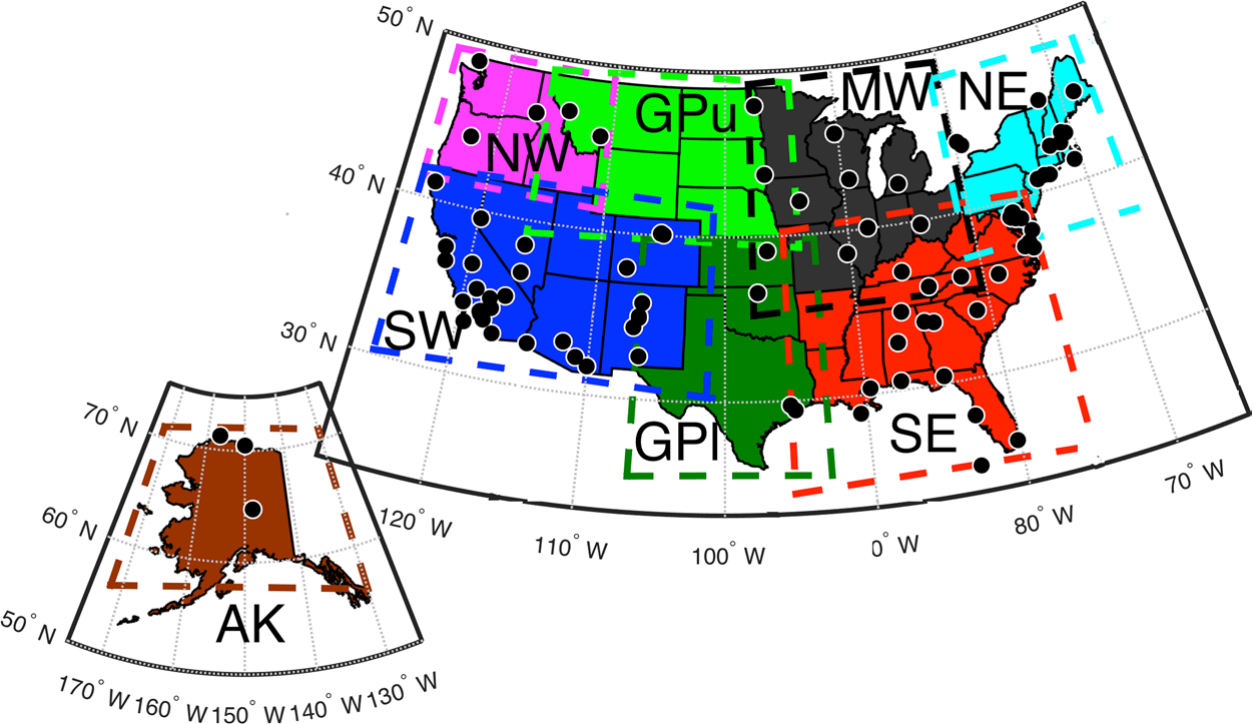
The eight U.S. National Climate Assessment (NCA) regions in which the aerosol-CIs are computed. The CIs are computed using MERRA-2 daily-averaged output from all grid cells within the dashed lines enclosing each region. Note the Great Plains region has been divided into two regions to ease interpretation of the analyses. Abbreviations: AK = Alaska, NW = Northwest, SW = Southwest, GPu = upper Great Plains, GPl = lower Great Plains, MW = Midwest, SE = Southeast, and NE = Northeast. Also shown within the map are the locations of AERONET sites from which data are presented in Figure S1. Figure was created using MATLAB (2016b; mathworks.com).

In order to diagnose and track changes in key observable properties of the climate system through time, a number of climate indicators (CI) have been developed and applied^14,15^. Many agencies that contribute to the U.S. Global Change Research Program (USGCRP) have developed and applied CIs to document and track changes in the physical, chemical, and anthropogenic (socio-economic) components of the climate system. The spatial or temporal resolutions of CIs vary widely: Some are global in scale while others are regional, and while some focus on the drivers of global change, others are more strongly focused on response variables. Existing USGCRP CIs thus include: Regional and global air temperature, precipitation, sea level, sea and land ice, and atmospheric concentrations of carbon dioxide, methane, nitrogen oxides, and fluorinated gases^14^. Despite the role of aerosols in perturbing regional climate, CIs of climate-relevant aerosol properties have yet to be developed^15^. Herein we propose a suite of aerosol-CIs, and illustrate how they are derived and applied using regions of the U.S. National Climate Assessment (NCA) program (Fig. 1). We demonstrate how these aerosol-CIs can be used to quantify variability and temporal trends in aerosol populations, and attribute changes through time to specific drivers of aerosol variability: Gaseous precursor and primary aerosol emissions, and meteorological conditions at the synoptic scale.

CIs must be predicated on high quality, uniform (gridded), and publically available data with well-defined provenance and an expectation that the variables on which they are based will continue to be measured into the future. Therefore observations, such as those from satellite- or ground-based remote sensing, are not suitable for deriving aerosol-CIs due to spatiotemporal discontinuities and a bias towards sampling cloud-free conditions^16^. Thus, we demonstrate the benefit of deriving the proposed aerosol-CIs from the first homogeneous, gridded reanalysis product that is constrained by satellite-based aerosol and meteorological measurements: Modern-Era Retrospective Analysis for Research and Application, Version 2 (MERRA-2)^17,18^. MERRA-2 provides gridded global hourly output of observable aerosol optical properties, including in cloudy-sky scenes, with high fidelity when evaluated relative to independent (non-assimilated) observations^17^.

Herein, we develop CIs of aspects of aerosol populations relevant for aerosol-radiation interactions and climate at the regional scale, and using output from MERRA-2 apply the aerosol-CIs to each NCA region (Fig. 1) to provide an illustrative example of how they can be used to quantify, characterize, and diagnose causes of historical trends in climate-relevant aerosol properties. To the first order, three key properties of the aerosol population determine the magnitude of the forcing due to aerosol-radiation interactions and thus the climate impact: Total columnar burden, size of the aerosols, and their composition^19^. Thus the aerosol-CIs we propose are based on: (1) AOD (550 nm), which is a measure of the column-integrated extinction of radiation and is approximately proportional to the aerosol mass concentration. (2) Ångström exponent (AE; 470–870 nm) which is qualitatively inversely proportional to particle size with a secondary dependence on aerosol composition. (3) Single scattering albedo (SSA; 550 nm) which is the ratio of scattering to total extinction, and describes the relative efficiency of radiation scattering (leading to an increase in the global albedo and cooling) by aerosols to radiation absorption (leading to atmospheric warming)^2^. As aerosols potentially impact regional scale climate in the U.S.^4–7,20^, the proposed aerosol-CIs are designed to characterize and track changes in regionally averaged mean conditions of these variables and their extreme values. Further aerosol forcing must occur on relatively large scale for an appreciable climate impact, and therefore the aerosol-CIs also characterize and track changes in the spatial scales of aerosol features (both spatial autocorrelation and scales of coherence) (see Methods).

## Results

### MERRA-2

The release of the MERRA-2 dataset constitutes the first real opportunity to develop and apply aerosol-CIs for the U.S. NCA regions, or any other part of the globe. Aerosol properties in the MERRA-2 reanalysis product are derived in part based on assimilation of AOD at 550 nm derived from remotely sensed properties such as spectral reflectances, solar and instrument geometry, cloud cover, and surface features into the Goddard Earth Observing System, version 5 (GEOS-5) model^18^ (see Methods). MERRA-2 has been subject to extensive evaluation relative to independent observations, and thus only limited additional evaluation was undertaken as part of this study and is focused on evaluation of the joint probabilities of the key variables considered herein: AOD, and AE and SSA relative to those from ground-based measurements of columnar aerosol properties from AErosol RObotic NETwork (AERONET) stations^21^ (see Methods; Figure S1).

### Development of aerosol-CIs

**Figure 2:**
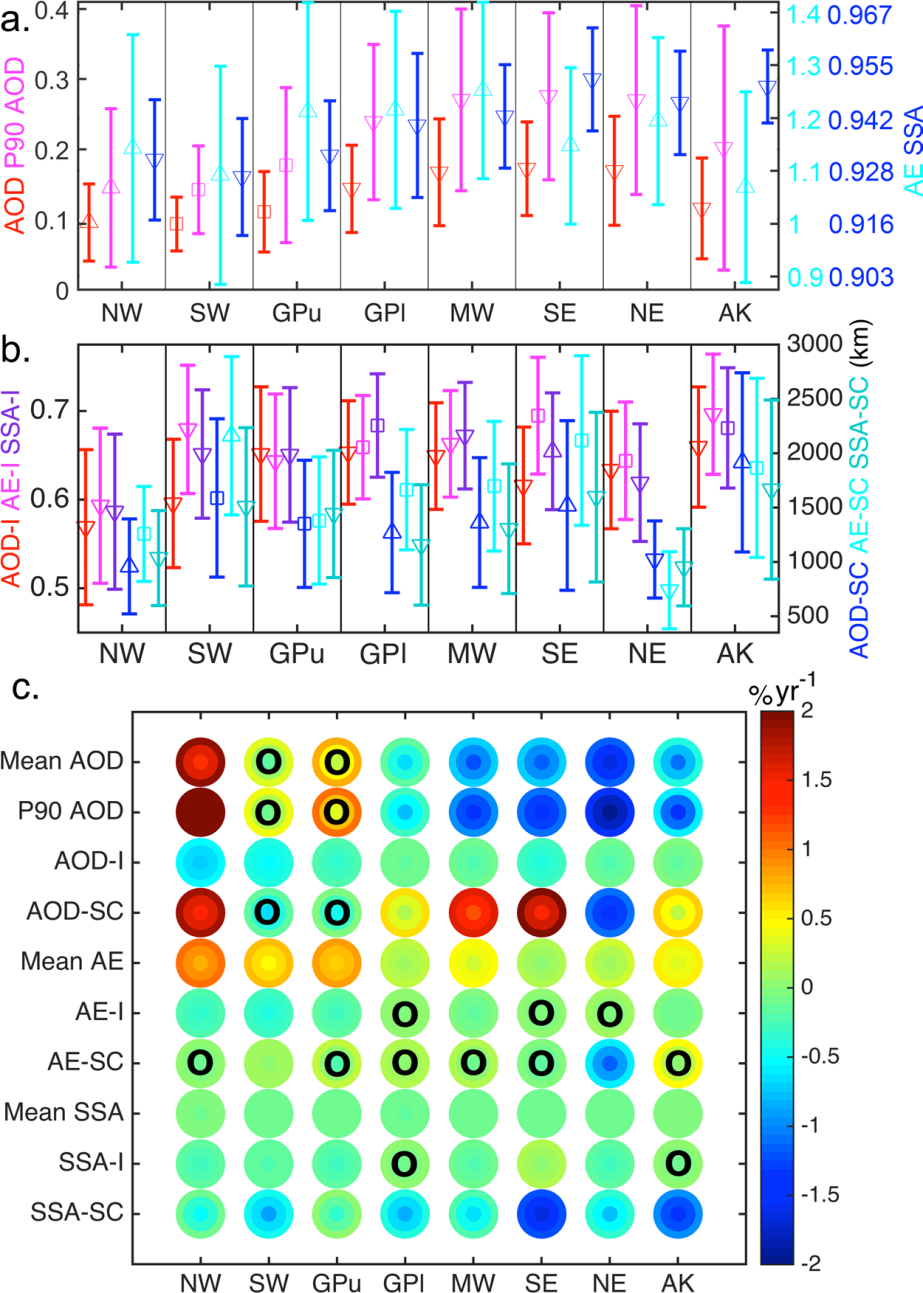
(**a**,**b**) Mean (marker) and ±1 standard deviation (whiskers) values of the aerosol-CIs during the study period (2000–2015). Upward and downward facing triangles indicate significant positive and negative trends as determined using Kendall’s tau-b, while square markers indicate no significant trend (at α = 0.05). (**c**) Percentage change per year in the CIs estimated using a linear regression fit (shown in Figures S2 and S3). The middle circles denote the normalized regression slopes (i.e. trends), and the inner and outer circles are the lower and upper bounds, respectively, of the 95% confidence intervals of these slopes. Black circles indicate trends that are not significant at α = 0.05.

AOD, AE, and SSA describe key aspects of aerosol particle populations that have greatest relevance to direct radiative forcing via aerosol-radiation interactions. Accordingly our proposed aerosol-CIs are based on daily values derived by averaging in space (i.e. over the NCA regional definitions shown in Fig. 1) and time, the hourly estimates of total column (anthropogenic and natural) AOD, AE, and SSA. The aerosol-CIs are thus daily mean AOD, AE, SSA and extreme (90^th^ percentile (P90 AOD)) AOD, along with two key metrics of the spatial patterns of these variables: The daily global spatial autocorrelation value (characterized using Moran’s-I^22^; AOD-I, AE-I, SSA-I) and the range of spatial coherence as derived using semivariograms^23^ of daily AOD, AE, and SSA fields within each region (AOD-SC, AE-SC, SSA-SC) (Fig. 2). Moran’s-I quantifies the degree of spatial clustering in the field and semivariograms quantify the distance at which two locations become independent. These ten aerosol-CIs are designed to track evolution of regional aerosol populations in terms of the overall aerosol columnar burden, average aerosol diameter, relative proportions of absorbing versus scattering aerosols, and the regional consistency of the spatial patterns of those properties.

Each aerosol-CI contains unique information about regional aerosol properties that have different implications for direct radiative forcing. These CIs also exhibit intra- and inter-annual variability and trends that are not consistent across indicators indicating the utility of all of the proposed aerosol-CIs to trend diagnostic and attribution analyses (Fig. 2). To detect potential redundancy in the aerosol-CIs, a principal component analysis (PCA) was conducted. Although the aerosol-CIs exhibit co-linearity, the aerosol-CIs tend to fall primarily on orthogonal principal components, and the PCA indicates that there is not a coherent, physically consistent set of synthetic, comprehensive indicators across the different regions. Further, for a true climate impact to be realized, aerosol radiative forcing must be expressed over a large area. Thus, there is a need to understand and quantify the degree to which climate-relevant aspects of aerosol populations are regionally coherent.

### Application of the aerosol-CIs to regions of the U.S. NCA

**Figure 3:**
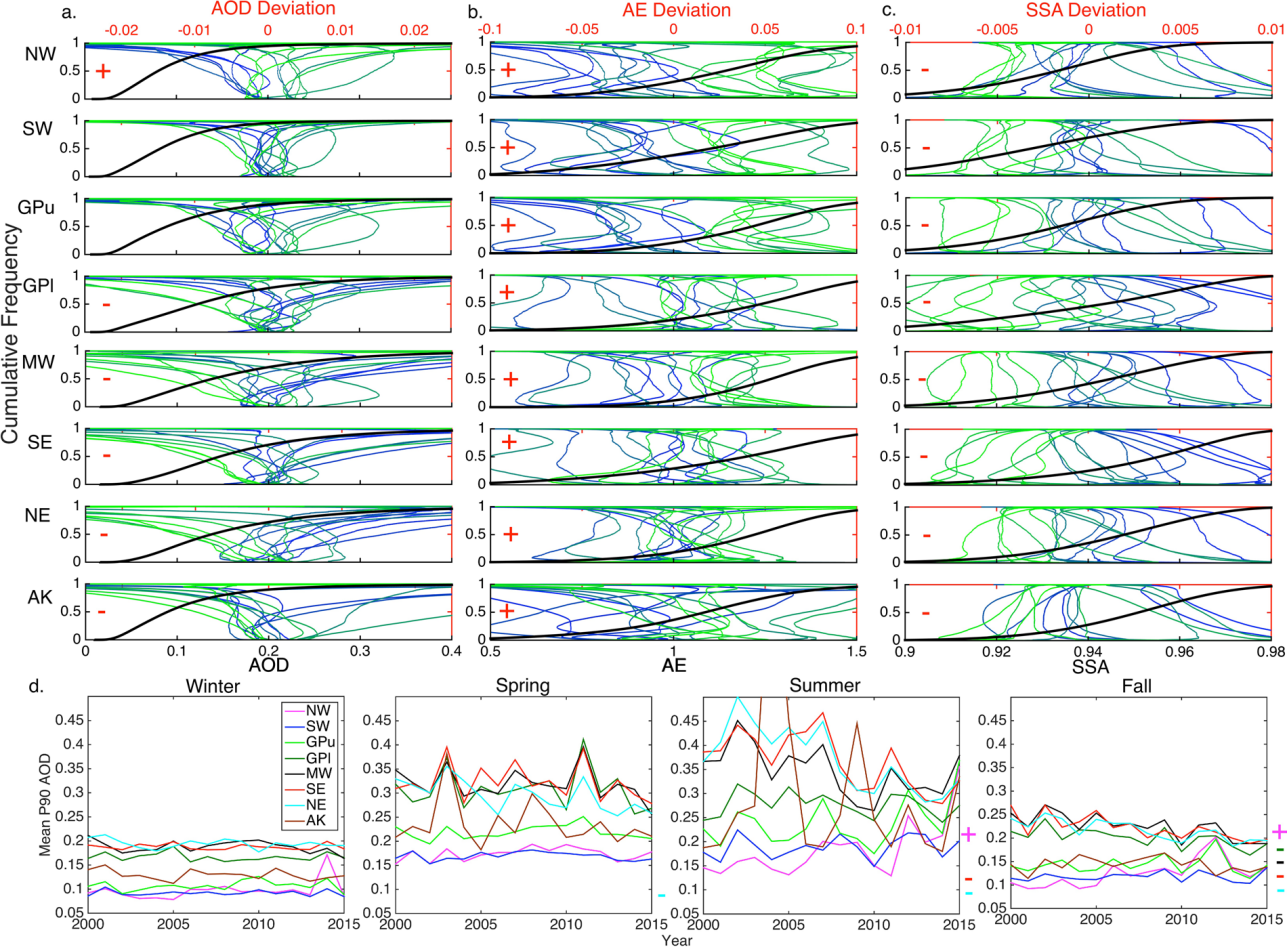
Cumulative distribution functions (cdf) of data from 2000–2015 for (**a**) AOD, (**b**) AE, and (**c**) SSA in each region. The cdf for all years is shown in black (labels under lower panel), while the deviation from the mean is shown for each year with the color scheme transitioning from blue (2000) to green (2015) (labels above top panel). (**d**) Time series of the yearly seasonal mean extreme AOD for each region. Significant trends in the daily mean values are indicated by a red ‘+’ or ‘−’ in each panel (a–c) for positive and negative trends, respectively, and to the right of each panel in (d).

Consistent with previous research, mean and extreme (P90) AOD declined in virtually all NCA regions over the period 2000–2015 (Fig. 2). Significant (hereafter α = 0.05, unless otherwise indicated) decreases are observed in five regions: the lower Great Plains (GPl), Midwest (MW), Southeast (SE), Northeast (NE), and Alaska (AK), but increased mean and extreme AOD is observed for the Northwest (NW), and there was no change in the Southwest (SW) and upper Great Plains (GPu). To examine trends in AOD, AE, and SSA across their respective probability distributions (c.f. to only mean and extreme values in the CIs), Fig. 3a–c shows the cumulative distribution functions (cdf) in each region for 2000–2015, as well as, the deviation from the mean cdf for each individual year. The direction of change and the presence of significant trends are consistent for mean and extreme (P90) AOD in all regions, but the magnitude of the change is larger for extreme AOD, indicating a narrowing of the AOD probability distributions (Fig. 3a). Significant regional AOD trends are ~1% year^−1^, while the magnitude of the extreme AOD trends are 1.2–1.4% year^−1^ in regions of decreasing AOD and 1.9% year^−1^ for the NW (Figs 2, 3, and S2). There is marked seasonality in some regions in terms of both the magnitude of and temporal trends in the aerosol-CIs. For example, extreme (P90) AOD significantly decreased in summer (the season of highest historical values), spring, and fall in NE, summer and fall in SE and MW (p-value = 0.06 for MW summer), and during fall in GPl. Conversely P90 AOD increased in summer and fall in NW (Fig. 3d).

The key utility of including two indices of spatial structure of the fields is illustrated by the divergent trends in these two aerosol-CIs. All regions exhibit decreased AOD spatial autocorrelation (AOD-I), but increased AOD spatial coherence (AOD-SC) is observed over the NW, GPl, MW, SE, and AK, and decreased AOD-SC is observed in the SW (p-value = 0.07), GPu (p-value = 0.15), and NE (Figures 2 and S3). Causes of these differences and the inter-annual variability in the aerosol-CI trends are discussed below.

Mean AE significantly increased across all eight regions, indicating a decrease in mean particle size (Figures 2 and S2). This shift to higher AE is observed across the probability distribution, implying a shift in fine mode aerosols to smaller sizes, as opposed to a relative increase in fine versus coarse mode aerosols (Fig. 3b). However, trends in the spatial metrics of AE are not uniform across the regions. Significant negative trends in AE-I are observed in NW, SW, GPu, MW, and AK (Figures 2 and S3), but only two regions exhibited significant changes in AE-SC and they showed different signs (increased in SW and decreased in NE). Thus, there is evidence that as the aerosol populations are, on average, decreasing in diameter at the regional scale, but there remain sub-regions within many of the NCA regions with high coarse mode concentrations (e.g., across all days, 50% of grid cells have AE ≤ 1.2 in the NW, SW, and GPu; Fig. 3b), possibly due to wind-blown dust events^24^.

Mean SSA and SSA-SC decreases are observed in all eight regions (Fig. 2). There are also decreases in SSA-I for all regions except SE where there were significant increases in SSA-I, although the significance of the trend is lower in GPl (p-value = 0.06) and AK (p-value = 0.16). It is noted that SSA is determined by the aerosol composition and the dynamic range of SSA in MERRA-2 is lower than observations^17,25^ (Figure S1); therefore the aerosol-CIs that relate to SSA must be viewed with caution in the current reanalysis product. However, these trends are consistent with a tendency towards a relatively more absorbing aerosol, thus reducing the net cooling from aerosols. Further, the trends in SSA-I and SSA-SC imply aerosol populations are becoming more spatially heterogeneous in terms of the relative contribution of absorption to total radiative extinction.

When applied to the U.S. NCA regions, the aerosol-CIs thus indicate substantial evolution of aerosol populations through time in ways that are relevant to regional climate forcing. Overall aerosol burdens have declined (2000–2015) and on average aerosol populations have changed to become more dominated by smaller diameter and more absorbing aerosols. They are also evolving in a way that causes a decrease in spatial autocorrelation, but increases in spatial coherence.

### Attribution of temporal trends in the aerosol-CIs

Attribution of observed trends in the aerosol-CIs, particularly deconvoluting changes resulting from changing anthropogenic emissions, natural emissions, and atmospheric conditions is critical to demonstrating the effectiveness of emission reduction policies, exploring and prioritizing potential climate change mitigation strategies, and making projections of possible future values of the aerosol-CIs. Thus, the aerosol-CIs for the NCA regions are examined below in the context of these key drivers of aerosol populations.

**Figure 4:**
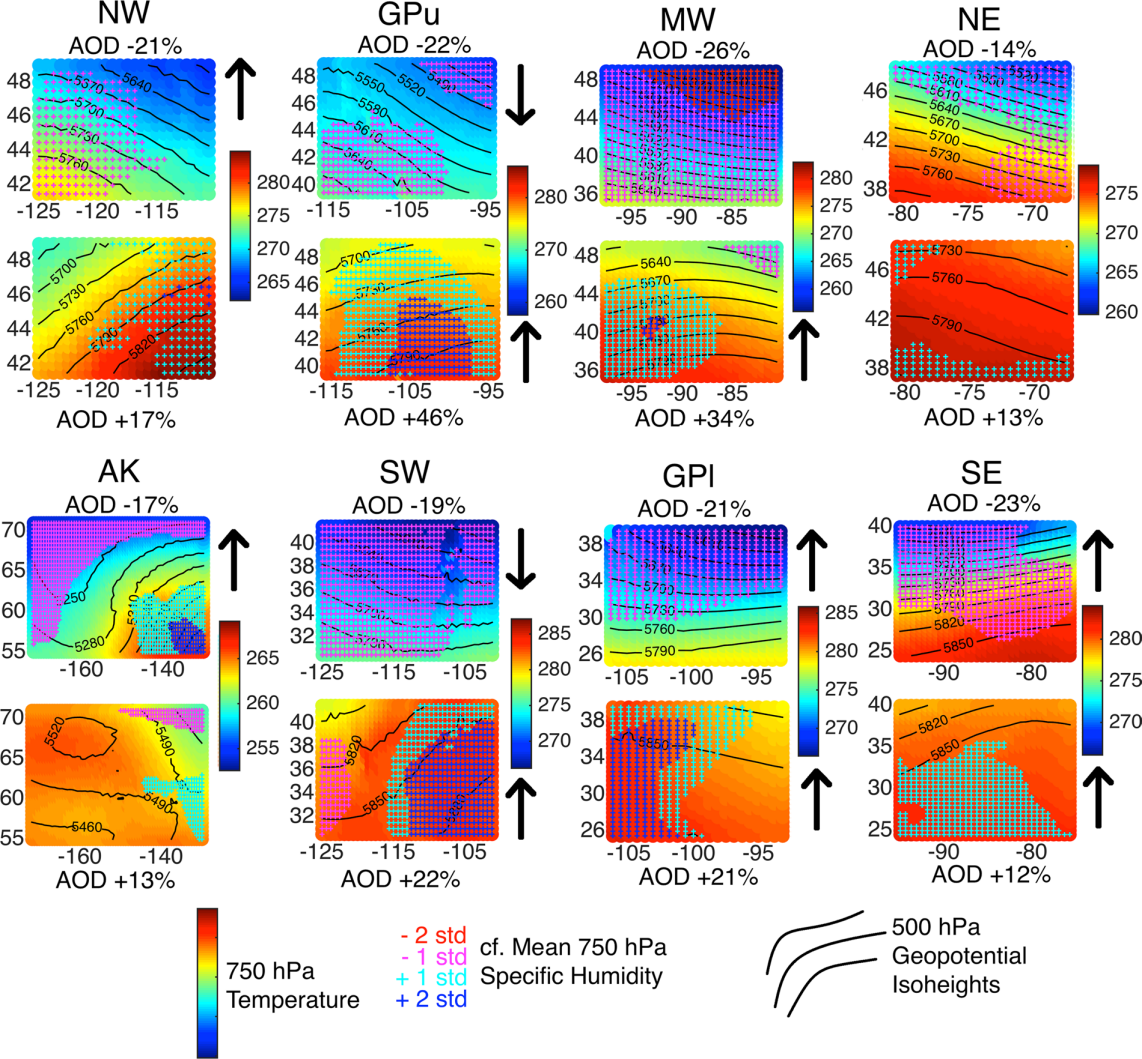
Mean synoptic conditions for synoptic types associated with anomalously low and high AOD for each region (locations shown in Fig. 1). The mean temperature at 700 hPa (in K) are shown by the background colors, the solid black lines depict the 500 hPa geopotential isoheights (in m), and the red, magenta, cyan, and blue stippling represent 700 hPa specific humidity anomalies −2, −1, +1, and +2 standard deviations from the mean for all days. The arrows beside the panels indicate the presence and direction of significant trends in the PC scores associated with these synoptic types. The abscissa and ordinate axes are longitude (degrees East) and latitude (degrees North), respectively.

Aerosol-climate interactions are reciprocal. Aerosols are a major driver of climate variability and change, but equally changes in climate alter aerosol concentrations and composition^26–28^. Further, previous research has illustrated a key role of synoptic scale meteorological conditions in determining regional aerosol concentrations under the current^3,29–31^ and possible future climate^32,33^. Consistent with that research, in each of the NCA regions, a number of synoptic types (i.e. repeated meteorological patterns) derived in a PCA of MERRA-2 meteorological output are associated with 10–20% AOD anomalies (positive and negative from the mean) (Fig. 4). The link to meteorological conditions at the synoptic scale is less pronounced for AE (the anomalies are <10%) and it appears SSA is relatively insensitive of the prevailing meteorological conditions (no synoptic type had a regionally average SSA anomaly of >2%). This finding re-emphasizes the complexity of aerosol populations and their related climate forcing, and highlights the importance of having multiple aerosol-CIs in order to fully characterize changes in climate-relevant aerosol properties.

Over all regions, synoptic types characterized by cooler (or milder) and drier conditions are associated with lower AOD. Conversely, anomalously high AOD is associated with warm and/or humid synoptic types, consistent with enhanced AOD under stagnant flow^29^ and aerosol growth by water uptake^34^. Over the northern and western regions of the contiguous U.S. (NW, SW, GPu, MW) southwesterly geostrophic flow is typically associated with positive anomalies in both mean and extreme AOD, while northwesterly flow is associated with negative anomalies in mean and extreme AOD (Fig. 4). Anomalously low AE in virtually all regions is often associated with cool, dry synoptic conditions, consistent with an increase in dust loading during dry conditions^24^. Conversely, high AE is associated with warm, humid conditions at the synoptic scale consistent with predominance of hygroscopic secondary aerosols.

Consistent with prior research that has indicated changes in global and regional temperature and humidity are likely to result in changing characteristics of the synoptic types^29,35^, the majority of synoptic types associated with large positive AOD anomalies in each region exhibit a significant positive trend in PC scores. Conversely, synoptic types associated with negative AOD anomalies exhibited trends that are divided between increasing and decreasing trends (Fig. 4). While there is evidence that some cool, dry days are also becoming cooler and drier, the dominant signal in this analysis is thus that synoptic types associated with elevated AOD are evolving to become more intense, i.e. warm, humid days becoming warmer and more humid. These changes in the synoptic-scale climate may thus partially offset emissions reductions^26,28^. While the intensity of the synoptic types has changed, the frequencies of individual synoptic types over each region do not exhibit significant temporal trends over the period 2000–2015.

**Figure 5:**
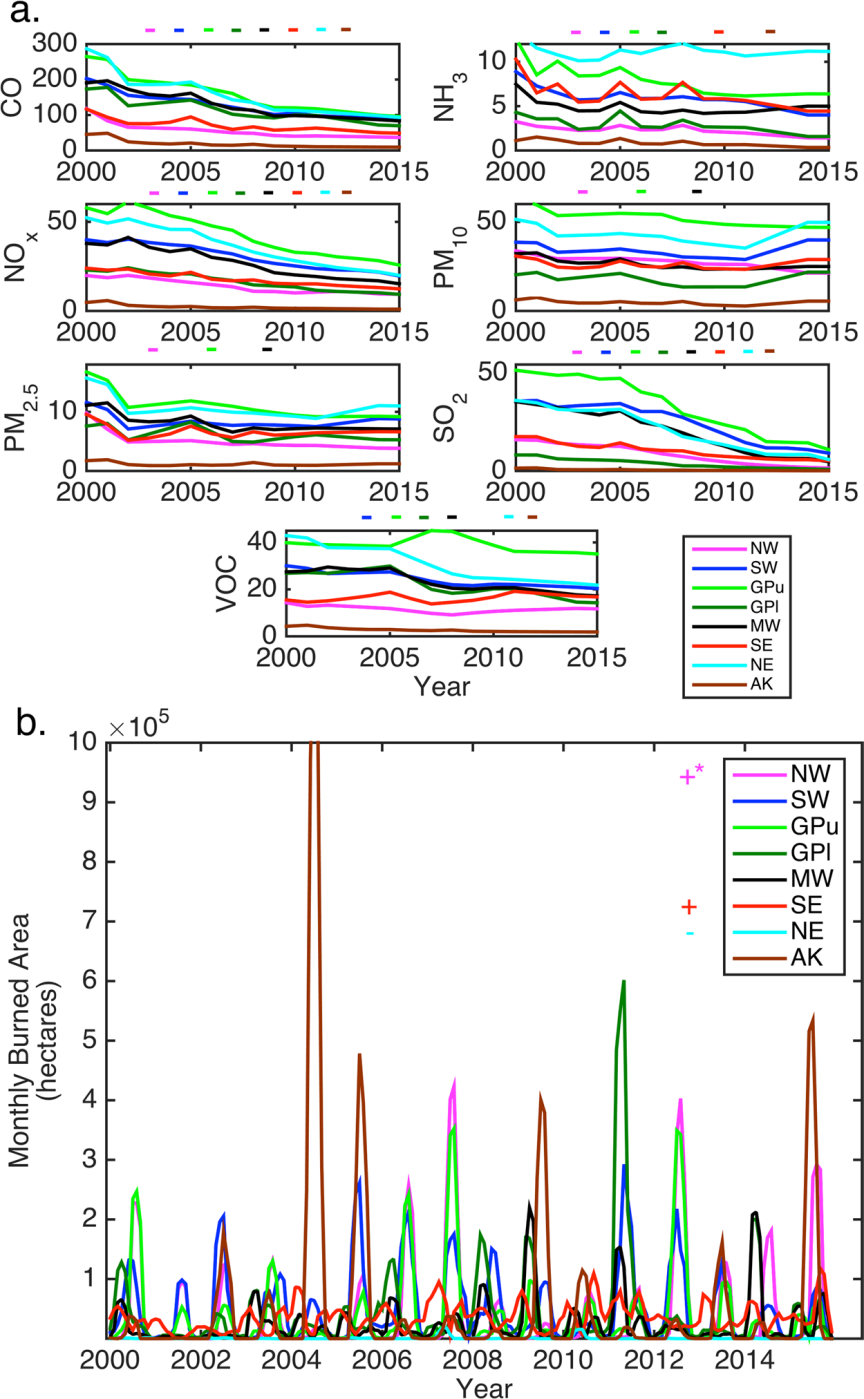
(**a**) Time series of annual anthropogenic emissions as reported in the U.S. EPA National Emissions Inventory of carbon monoxide (CO), ammonia (NH_3_), nitrogen oxides (NO_x_), particulate matter <10 μm (PM_10_), fine particulate matter <2.5 μm (PM_2.5_), sulfur dioxide (SO_2_), and volatile organic compounds (VOC) by region, in thousands of tons per year^36^. (**b**) Time series of wildfire occurrence expressed as monthly burned area for each region, derived from MODIS measurements^48^. The sign of significant trends are shown above each panel in (a) and next to the legend in (**b**) (*positive trend in NW monthly burned area p-value = 0.13).

Consistent with policy enacted under the U.S. Clean Air Act that has resulted in declining anthropogenic pollutant emissions over the study period, regionally integrated emissions of key aerosol precursor species, sulfur dioxide (SO_2_) and nitrogen oxides (NO_x_), exhibit a significant negative trend for all eight NCA regions over the period 2000–2015. Further ammonia (NH_3_) emissions exhibit a negative trend in all regions except the MW and NE, and volatile organic compounds (VOC) emissions exhibit a negative trend in all regions except the NW and SE (Figure 5)^36^. Consistent with this, mean and extreme AOD significantly decreased in GPl, MW, SE, and NE, and seasonal extreme AOD decreased in the fall in GPl, summer and fall in MW and SE, and spring, summer, and fall in NE. The overall tendencies in aerosol-CIs, including the significant decrease in mean and extreme AOD over GPl, MW, SE, and NE, are thus consistent with a decrease in sulfate aerosol abundance due to the reduction in SO_2_ emissions (e.g., correlation coefficients between annual SO_2_ emissions and extreme summer (except GPl) and fall AOD are >0.57 over these regions). Congruent with this decline in SO_2_ emissions, the annual deviations from the overall cumulative distribution functions (cdf) imply that almost the entire probability distribution of AOD has shown a shift towards lower values (Fig. 3a). Further, because sulfate has a high SSA (near unity)^37^, a reduction in secondary sulfate aerosol would also contribute to the observed decline in regionally-averaged SSA. Reduced production of sulfuric acid may also lead to a reduction in mean aerosol diameter, implied by the increase in AE, due to a reduction in condensational growth. While historic trends in black carbon (BC) emissions are highly uncertain (e.g., from biomass burning), it is estimated emissions from mobile sources, the largest BC source in the U.S., decreased by 32% from 1990–2005 (ref.^38^). Further, BC only contributes to ~4% of global AOD^18^. Thus changes in SSA are likely not due to changes in anthropogenic BC emissions. Secondary organic aerosols are also a substantial component of aerosol mass and AOD over much of the eastern U.S.^39^. Thus an additional contributory factor to declining AOD in these regions is the reduction in anthropogenic VOC emissions and secondary organic aerosol formation. Accordingly, the correlation coefficients between annual VOC emissions and extreme summer and fall AOD in the NE and MW are >0.61. Thus, consistent with prior research, historical temporal trends of AOD across much of the contiguous U.S. are strongly responsive to emission reductions associated with the Clean Air Act.

Despite reductions in anthropogenic aerosol precursor gas emissions, it is worthy of note that primary aerosol emissions exhibit a significant trend only in the NW, GPu, and MW (Fig. 5), and that biogenic VOC, dust, and wildfire emissions exert a substantial impact on aerosol burdens and optical properties^40,41^. For example, there is a clear peak in extreme AOD in the spring of 2011 in the GPl, MW, and SE when wildfire burned area in the GPl was approximately four times greater than any other year (Figs 3 and 5). In the GPl, the lack of association (i.e. lower correlation coefficients) between annual anthropogenic emissions and extreme AOD in three of the four climatological seasons and the observed decreased SSA may also be in part due to increased abundance of dust aerosols, consistent with remote sensing measurements that indicate increased dust-related absorption aerosol optical depth (AAOD) over the central U.S.^24^. The declining trend in AOD in AK is also not very strongly linked to changes in anthropogenic emissions, but there is a significant positive association between extreme summer AOD and wildfire burned area (r = 0.96). This is clearly evident in 2004, 2009, and 2015, when positive excursions in monthly burned area (Fig. 5) coincide with spikes in summer extreme AOD (Fig. 2).

Only the NW region exhibits a significant positive trend in annual mean AOD, with extreme AOD increasing in the summer and fall (Figs 2 and 3). This is despite declines in regional anthropogenic emissions (Fig. 5), and may reflect confounding influences from increased wildfires (seasonal burned area and extreme AOD in summer and fall exhibit co-variability with r = 0.53 and 0.75, respectively) and long-range transport. For example, Siberian fires in the summer of 2012 impacted air quality in the Pacific NW^41^, and are evident in high P90 AOD during the 2012 summer and fall (Fig. 2).

The decrease in the spatial autocorrelation in AOD (Figures 2 and S3) along with the decreased anthropogenic aerosol precursor emissions in each region (Fig. 5) indicates an increasing influence of local sources on sub-regional aerosol concentrations and thus increased grid cell–to–grid cell variability in aerosol populations. Conversely, scales of spatial coherence (distance at which grid cells become independent) are increasing, which may be linked to changes in synoptic scale conditions (Fig. 4). High and low AOD are generally associated with warm, humid and cool, dry conditions, respectively. The positive trend in PC scores for synoptic types associated with high positive AOD anomalies indicate a tendency towards intensification of meteorological conditions associated with large direct aerosol radiative forcing that may be offsetting some of the effects of emission controls. As climate conditions continue to evolve, this highlights the critical need to better understand the feedbacks between climate and aerosol populations.

## Discussion

Use of climate indicators to represent key components of the climate system is an increasing focus of the U.S. NCA. For this reason, we advocate that aerosol-CIs are urgently needed to track a key aspect of the radiation balance of Earth, air quality, and biogeochemical cycles, and that aerosol-CIs should be generated and interpreted at the regional scale. The guidance for developing CIs is that they should be relatively straightforward to compute and readily evaluated in both the contemporary and possible future climate. Thus, the aerosol-CIs we propose can be readily derived for any gridded data set and therefore can be applied to any region using current and future generation reanalysis products and/or output from regional and climate models.

The aerosol-CIs presented herein are designed to be useful in tracking changes in climate relevant aspects of the aerosol population and to assist in diagnosing the causes of changes in aerosol populations at the regional scale. Their utility in the former regard is illustrated by application to the NCA regions, and specifically the finding that mean and extreme AOD and SSA is declining and AE is increasing over most of the U.S. consistent with a tendency towards lower aerosol burdens that are increasingly dominated by smaller diameter and relatively more absorbing aerosols. This implies a decline in the degree to which aerosols have offset greenhouse gas related warming of the climate over much of the contiguous U.S.

The aerosol-CIs are also defined using two geospatial metrics: Spatial correlation and spatial coherence. The former (Moran’s I) characterizes normalized co-variability and is a measure of the degree to which daily fields of AOD, AE, and SSA exhibit spatial clustering. The latter is a measure of the distance (range in the semivariogram) at which spatial fields become independent, and thus the extent to which the aerosol forcing can impact regional climate. The utility of these two spatial metrics in terms of diagnosing causes of changes in aerosol populations at the regional level is also indicated by the presence of divergent trends in AOD-I and AOD-SC in the NCA regions. These findings imply a tendency towards more grid cell–to–grid cell variability in aerosol populations, due to declining regional precursor and aerosol emissions leading to an increase in the relative importance of local emissions, within larger areas of increased spatial coherence (i.e. large range values from the semivariograms) in part due to an increase in the intensity of the predominant modes of synoptic scale meteorology.

Future work is needed to examine aerosol trends in global regions outside of the U.S. that are characterized by markedly different emissions and climate trends. Additionally, analyses of reanalysis products are only as good as the assimilation data and model used to develop the product. Thus the CIs should be applied to future reanalysis products that assimilate improved bias-correction assimilated data, data from additional, recently launched sensors, and more sophisticated model frameworks with improve aerosol treatment and emissions inventories.

## Methods

### MERRA-2

MERRA-2 is derived using assimilation of both meteorological and aerosol observations every 6 and 3 hours, respectively, into the Goddard Earth Observing System, version 5 (GEOS-5) model^18^. It provides hourly, global gridded output of meteorological variables and aerosol optical properties including AOD, AE, and aerosol scattering extinction at 0.625° by 0.5° resolution. The aerosol characteristics are constrained using a wide suite of remote sensing products. For example, AOD at 550 nm is derived from Moderate Resolution Imaging Spectroradiometer (MODIS) measurements on both the Terra and Aqua satellites (Collection 5)^42^ of reflectances, solar and instrument geometry, cloud cover, and surface features^18^ using a neural network retrieval (NNR) trained using AERONET measurements. A similar approach is used to assimilate Advanced Very High Resolution Radiometer (AVHRR)^43^ measurements of radiances, total precipitable water, wind speed, and solar and instrument geometry trained to the MODIS NNR. MISR AOD is assimilated only over bright surfaces^44^, and ground-based AOD measurements from the AERONET^21^ are assimilated after 1999. As the density of assimilated aerosol optical properties and meteorological measurements increases greatly after 2000 (refs^18,45^), the analysis presented here is limited to 2000–2015.

MERRA-2 output includes surface short- and longwave radiation fluxes, with and without clouds, and with and without aerosols, which could be used to estimate aerosol radiative forcing. However these properties are dependent on the radiative transfer model and treatment of aerosol optical properties within the reanalysis model. Thus, herein we only use observable variables that are more closely tied to the assimilated data.

MERRA-2 aerosol properties that are not directly assimilated have been compared to, and found to be in reasonable agreement with, satellite-based radiometric measurements. For example, monthly mean biases relative to the Ozone Monitoring Instrument (OMI) retrieved absorption aerosol optical depth (AAOD) are typically < |0.02| over the NCA regions, and MERRA-2 reproduces the aerosol vertical profile (e.g., height of peak attenuation backscatter) retrieved from the Cloud-Aerosol Lidar with Orthogonal Polarization (CALIOP) over the continental U.S. (CONUS)^17^. MERRA-2 has also been evaluated relative to near-surface measurements of PM_2.5_. Again the results indicate a relatively high degree of consistency with independent observations. For most months across the CONUS, MERRA-2 PM_2.5_ is within one standard deviation of the *in situ* measurements, although there is an underestimation of winter PM_2.5_ concentrations over the northwest and northeast U.S., potentially due to lack of nitrate aerosols in MERRA-2 (ref.^17^).

Our analysis of the joint probabilities of AOD, and AE and SSA from MERRA-2 relative to AERONET, indicate good agreement, although MERRA-2 underestimates the dynamic range of AE and SSA (Figure S1). Such underestimation is common when comparing gridded aerosol datasets that represent area means (~2,500 m^2^ for MERRA-2) versus *in situ* observations such as the pseudo-point measurements from AERONET. MERRA-2 reproduces the observed region-to-region variability in aerosol radiative properties and the MERRA-2 versus AERONET differences tend to be smaller than region-to-region differences (Figure S1).

Physical variables from MERRA-2 used here within the synoptic-scale meteorological classification have also been extensively evaluated in the previous MERRA release. For example, the mean residual between MERRA and observations is <0.5 hPa for Northern hemisphere surface pressures and ~<1 K for temperature through the depth of the atmosphere relative to radiosonde measurements^46^. Since the original MERRA reanalysis, the GEOS model has been further updated to reduce erroneous trends and discontinuities deriving from breaks in assimilated measurements, and to reduce biases in the water cycle. For all regions in the CONUS, MERRA-2 mean summer precipitation is within ~0.5 mm day^−1^ (~0.1–0.2 mm day^−1^ averaged across the CONUS) of surface rain gauge measurements and exhibits an anomaly correlation of ~0.9 for 1980–2011 (ref.^47^).

The advantages of using the MERRA-2 product for development of aerosol-CIs are manifold. These include use of a consistent data assimilation system for the entire period of record. However, any reanalysis system is subject to inherent uncertainties due either to assimilated variables and/or the model system. For example, an artificial trend exists in Terra radiances assimilated into MERRA-2, which may confound the trend analysis presented herein. Thus trends identified here should be further validated with future MERRA releases in which this trend is corrected and/or with other aerosol reanalysis products as they become available.

### Wildfire and anthropogenic emissions

Estimates of wildfire occurrence and spatial extent used herein to diagnose trends in the aerosol-CIs derive from the Global Fire Emissions Database (GFED4) monthly burned area product. GFED4 provides monthly estimates of hectares of burned area on a 0.25° grid derived from the MODIS (Collection 5.1) monthly burned area product^48^.

Annual estimates of anthropogenic emissions of carbon monoxide (CO), NH_3_, NO_x_, PM_10_, PM_2.5_, SO_2_, and VOCs are also used in attribution of changes in the aerosol-CIs. These estimates are accumulated for all states within each of the NCA regions and derive from the EPA’s state level National Emissions Inventory (NEI)^36^. It is noted that there is inherent uncertainty in emissions estimates due to spatiotemporal variability in emission sources, measurement and sampling errors, and the simplification of modeled emissions processes. For example, SO_2_ emissions rely on the sulfur content of the combustible material, biogenic emissions vary with environmental conditions, and NH_3_ emissions lack wide-spread regulatory restrictions and ambient NH_3_ measurements are scarce^49,50^. Additionally, MERRA-2 aerosol speciation depends, in part, on the magnitude of prescribed emissions, which do not evolve (i.e. persistency is assumed) during the later years of the study period^18^. Despite these uncertainties, measurements of species important for secondary aerosol formation, e.g. SO_2_, suggest that trends in emissions are robust^13,51^.

### Statistical methods used to derive and interpret the aerosol-CIs

The aerosol-CIs we propose quantify the regionally-averaged mean AOD, AE, and SSA; extreme (90^th^ percentile) AOD; and two geostatistical metrics of spatial autocorrelation and spatial coherence of AOD, AE, and SSA. The regionally averaged mean and P90 values are computed from hourly output that are aggregated in space and time to generate daily mean values for each property that then comprise each CI. While a spatial mean is used here, previous work indicates that spatiotemporal averages are sensitive to averaging methodology^52^, particularly for variables such as AE^53^. The spatial autocorrelation (AOD-I, AE-I, SSA-I) and spatial coherence (AOD-SC, AE-SC, SSA-SC) statistics are computed from the daily mean of the hourly output for each grid cell.

The global spatial autocorrelation for each region and aerosol parameter is computed at the daily timescale and quantified using Moran’s I^22^:

1 $$I=\frac{N}{{\sum }_{i=1}^{N}{\sum }_{j=1}^{N,i\ne j}{w}_{ij}}\frac{{\sum }_{i=1}^{N}{\sum }_{j=1}^{N,i\ne j}{w}_{ij}({X}_{i}-\bar{X})({X}_{j}-\bar{X})}{{\sum }_{i=1}^{N}({X}_{i}-\bar{X})}$$

2 $${w}_{ij}=\frac{1}{{{D}_{ij}}^{2}\,}\frac{1}{{\sum }_{i=1}^{N}{\sum }_{j=1}^{N,i\ne j}\frac{1}{{{D}_{ij}}^{2}}}$$

where N is the number of grid cells, w_ij_ is the weight for grid cells i and j, X_i_ is the daily mean value (AOD, AE, or SSA) at grid cell i, $\bar{{\rm{X}}}$ is the mean of the daily means for all grid cells, and D_ij_ is the great circle distance between the centroid of grid cell i and j. Values approaching 1 and −1 indicate positive and negative spatial autocorrelation, respectively, while 0 indicates a random spatial field. Significance for rejecting the null hypothesis of no spatial autocorrelation is determined by calculating a z-score for each I:

3 $$Z=\frac{I-E(I)}{Var(I)}$$

4 $$E(I)=-\frac{1}{N-1}$$

5 $$Var(I)=\frac{N{S}_{4}-{S}_{3}{S}_{5}}{(N-1)(N-2)(N-3){({\sum }_{i=1}^{N}{\sum }_{j=1}^{N,i\ne j}{w}_{ij})}^{2}}-E{(I)}^{2}$$

6 $${S}_{1}=\frac{1}{2}\sum _{i=1}^{N}\sum _{j=1}^{N,i\ne j}{(2{w}_{ij})}^{2}$$

7 $${S}_{2}=\sum _{i=1}^{N}{(2\sum _{j=1}^{N,i\ne j}{w}_{ij})}^{2}$$

8 $${S}_{3}=\frac{\frac{1}{N}{\sum }_{i=1}^{N}{({X}_{i}-\bar{X})}^{4}}{{(\frac{1}{N}{\sum }_{i=1}^{N}{({X}_{i}-\bar{X})}^{2})}^{2}}$$

9 $${S}_{4}=({N}^{2}-3N+3){S}_{1}-N{S}_{2}+3{(\sum _{i=1}^{N}\sum _{j=1}^{N,i\ne j}{w}_{ij})}^{2}$$

10 $${S}_{5}=({N}^{2}-N){S}_{1}-2N{S}_{2}+6{(\sum _{i=1}^{N}\sum _{j=1}^{N,i\ne j}{w}_{ij})}^{2}$$

The spatial coherence of each variable in each region is computed using semivariograms which describe the semivariance as a function of separation distance between all grid cell pairs^23^:

11 $$\gamma (h)=\frac{{\sum }_{i=1}^{N,i\in Q}{\sum }_{j=1}^{N,{D}_{ij}\in h}{[{X}_{i}-{X}_{j}]}^{2}}{N(h)\times |Q|}$$

where N(h) is the number of grid cell pairs that are separated by a great circle distance of h, X_i_ and X_j_ are the daily mean values (AOD, AE, or SSA) at grid cells i and j, respectively, h is a bin range of separation distances, and Q is the set of all grid cells not within three grid cells of the domain border. The empirical semivariogram fit, γ(*h*), is binned in 100 km increments (i.e. ${\rm{\gamma }}(\,1\mbox{--}100\,\mathrm{km})$ includes all grid cell pairs separated by 1–100 km). An exponential fit is used to model γ(*h*) assuming an exponential decay in correlation with distance and for physical interpretability of the model^53,54^.

12 $$\gamma ^{\prime} (h)={C}_{n}+{C}_{p}(1-{e}^{-\frac{3h}{a}})$$

where γ′(*h*) is the exponential model fit; C_n_ is the nugget describing the semivariance at zero spatial lag, resulting from variability at scales below data resolution^54^; C_p_ is the partial sill, where the sill, C_n_ + C_p_, is the semivariance as h $\to$ ∞; and a is the range or distance at which 95% of the sill is reached, indicating the distance at which two locations are no longer correlated. γ(*h*) is calculated for each day, and γ′(*h*)is fit to the mean γ(*h*) for all days in each season^53^. For the CIs to be tracked through time, a single daily quantity is required. Thus, the daily “scale of spatial coherence”, SC, is herein defined as the minimum h where γ(*h*)> 0.75 × C_p_(a_s_), where C_p_(a_s_) is the partial sill for that season. While the spatial structure of the AOD and SSA fields is well represented by an exponential model, within the spatial extent of the individual regions AE semivariance tends to increase linearly with distance leading to higher uncertainty in a range determined using the exponential semivariogram model.

Temporal trends in the aerosol-CIs are quantified and the significance determined using Kendall’s tau-b (τ_b_) rank coefficient^55^. τ_b_ is calculated by comparing all pairs of observations, {(t_i_, X_i_), (t_j_, X_j_)} where X_i_ and X_j_ are the variable (AOD, AE, SSA) at time t_i_ and t_j_, respectively:

13 $${\tau }_{b}=\frac{C-D}{\sqrt{[\frac{N(N-1)}{2}-{\sum }_{i=1}^{N}\frac{t{x}_{i}(t{x}_{i}-1)}{2}][\frac{N(N-1)}{2}-{\sum }_{i=1}^{N}\frac{t{t}_{i}(t{t}_{i}-1)}{2}]}}$$

14 $$C-D=\sum _{i=1}^{N}\sum _{j=i+1}^{N,j\ne i}\{\begin{array}{c}\,if\,[sign(({X}_{i}-{X}_{j})({t}_{i}-{t}_{j})) > 0]=1\\ \,if\,[sign(({X}_{i}-{X}_{j})({t}_{i}-{t}_{j})) < 0]=-1\\ else=0\end{array}\}$$

15 $$t{x}_{i}=|X:\,X={X}_{i}|$$

16 $$t{t}_{i}=|t:t={t}_{i}|$$

where N is the number of observations. τ_b_ > 0 indicates a positive trend and τ_b_ < 0 indicates a negative trend. The significance of the trend is quantified using z-scores^56^:

17 $$Z=\frac{C-D}{\sqrt{\frac{{v}_{o}-{v}_{x}-{v}_{t}}{18}+{v}_{1}+{v}_{2}}}$$

18 $${v}_{0}=N(N-1)(2N+5)$$

19 $${v}_{x}=\sum _{i=1}^{N}t{x}_{i}(t{x}_{i}-1)(2t{x}_{i}+5)$$

20 $${v}_{t}=\sum _{i=1}^{N}t{t}_{i}(t{t}_{i}-1)(2t{t}_{i}+5)$$

21 $${v}_{1}=\frac{{\sum }_{i=1}^{N}t{x}_{i}(t{x}_{i}-1){\sum }_{j=1}^{N}t{t}_{j}(t{t}_{j}-1)}{2N(N-1)}$$

22 $${v}_{2}=\frac{{\sum }_{i=1}^{N}t{x}_{i}(t{x}_{i}-1)(t{x}_{i}-2){\sum }_{j=1}^{N}t{t}_{j}(t{t}_{j}-1)(t{t}_{j}-2)}{9N(N-1)(N-2)}$$

The slope of the trends, in terms of percentage change per year, is estimated to be the slope of a linear regression fit to the CIs’ time series.

It is hypothesized that changes in anthropogenic and natural precursor and primary aerosol emissions will be associated with changes in the aerosol populations. The significance of this association is quantified using the Pearson’s r correlation coefficient.

Prior research indicates that synoptic meteorological conditions are also a key control of aerosol concentrations^29,30^. Thus, PCA is used to derive a daily synoptic classification for all days in the study period and investigate the interaction between synoptic conditions and aerosol properties, and to determine the impact of meteorology on the CIs trends. Predictors used in the PCA are air temperature and specific humidity at 700 hPa plus 500 hPa geopotential heights from MERRA-2. The number of PCs to retain for each region was determined using a scree test^57^ and the retained factors are rotated using a Varimax rotation^58^. Between six and nine components (i.e. unique synoptic types) were retained for each of the eight NCA regions. The PC scores for each day (i.e. similarity to the major modes of variability as characterized by the PCs) are used to track changes in the frequency of each synoptic type (i.e. counts of days with highest similarity to each of the modes) and the intensity of the types (i.e. the magnitude of the scores for each PC). The mean anomaly of each aerosol-CI on all days classified by each synoptic type, calculated relative to the mean aerosol-CI computed for all days, is used to illustrate the importance of meteorological conditions at the synoptic (regional) scale in determining aerosol properties.

### Data availability

MERRA-2 data is available from the Goddard Earth Science Data and Information Services Center (https://disc.sci.gsfc.nasa.gov/), AERONET data is available from https://aeronet.gsfc.nasa.gov/, GFED4 is available from http://www.globalfiredata.org/, and NEI is available from https://www.epa.gov/sites/production/files/2016–12/state_tier1_90–16.xls.

## Electronic supplementary material

Supplementary Information
