# Supplementary material for: Developing and diagnosing climate change indicators of regional aerosol optical properties
Source: Sci Rep. 2017 Dec 22;7:18093. doi: 10.1038/s41598-017-18402-x (PMC5741728; doi:10.1038/s41598-017-18402-x)
Supplement: Supplementary file 1 — Supplementary Information [file 41598_2017_18402_MOESM1_ESM.pdf]

Supporting Information for

Developing and diagnosing climate change indicators of regional aerosol optical properties

Ryan C. Sullivan[a+*], Robert C. Levy[b], Arlindo M. da Silva[b], and Sara C. Pryor[a,c]

a. Department of Earth and Atmospheric Sciences, Cornell University, Ithaca, NY

b. NASA Goddard Space Flight Center, Greenbelt, MD

c. Pervasive Technology Institute, Indiana University, Bloomington, IN

+ Now at Environmental Science Division, Argonne National Laboratory, Argonne, IL, USA

**Corresponding author:*

rcsullivan@anl.gov

Contents of this file

Figure S1, S2, and S3

Introduction

This supporting information provides figures comparing the joint probability distributions of AOD, and AE and SSA from MERRA-2 and AERONET, and illustrating the trends in the aerosol climate indicators.

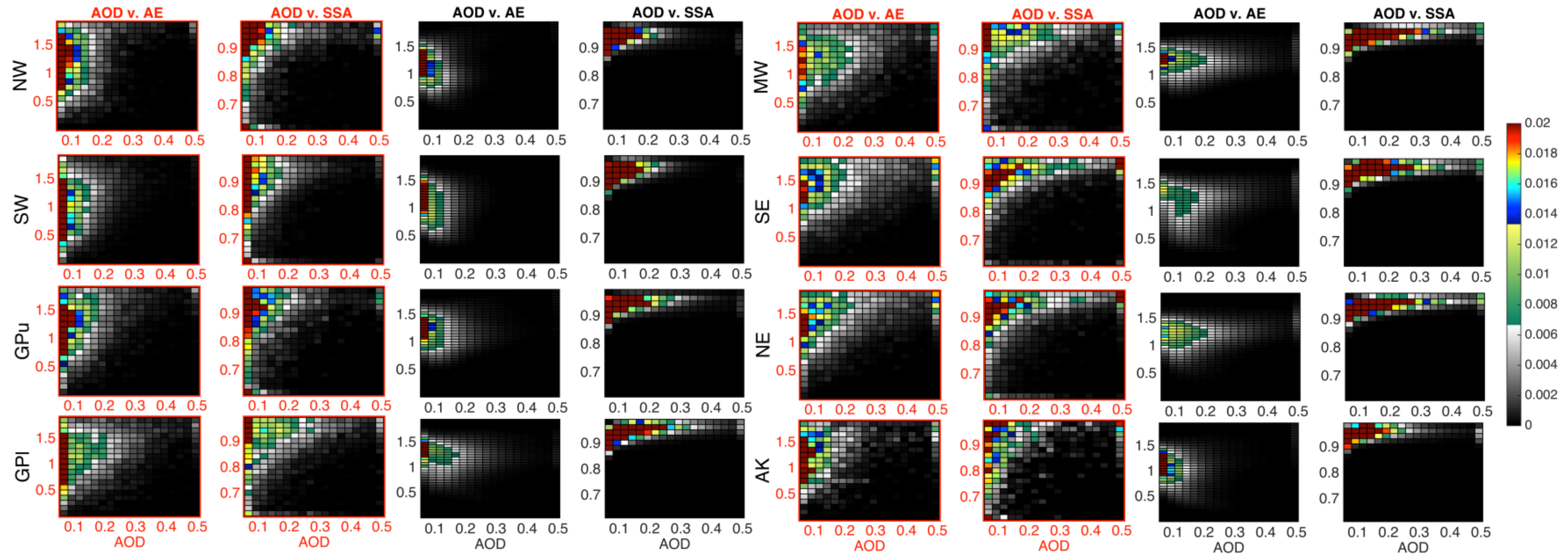

Figure S1. Joint probability distributions (jpd) for the daily mean aerosol optical properties in all grid cells in each region from MERRA-2 (black frame and labels) and all stations in each region from AERONET (red frame and labels). The jpd for AERONET include only stations with > 1 year of data and data from all months (except for in Alaska where no data are available in winter). Locations of AERONET stations are shown in Figure 1. In all panels AOD is shown on the abscissa axis, while the AE and SSA are shown in the ordinate axis. AOD is at 550 nm, SSA is at 550 nm and 440 nm, and AE is at 470 – 870 nm and 440 – 675 nm, respectively for MERRA-2 and AERONET. Note: As AERONET retrievals are assimilated into MERRA-2, the two datasets are not independent.

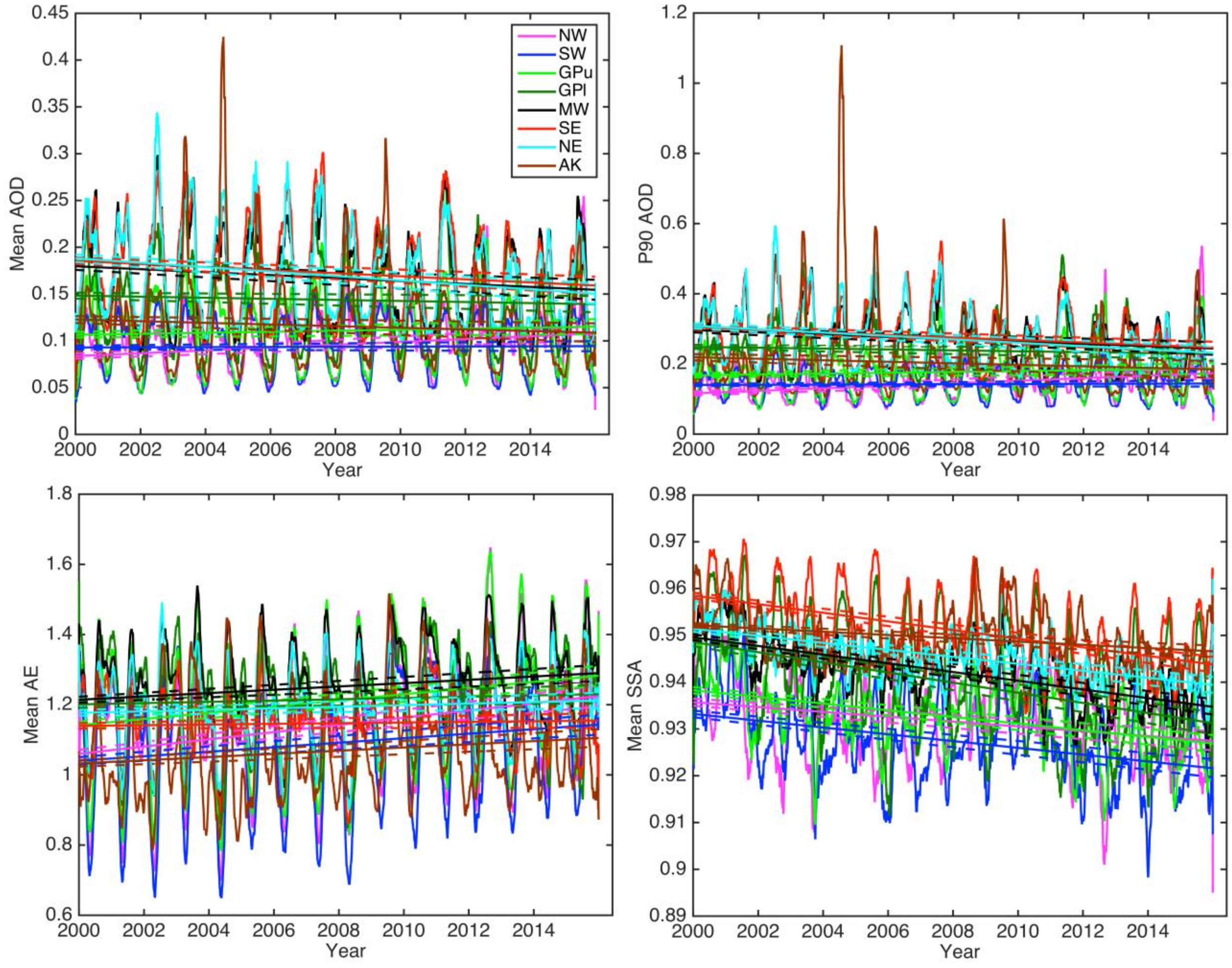

Figure S2. Trends in mean AOD, AE, and SSA, and extreme AOD. The time series has been smoothed in the plot using a 50-day running mean for legibility. The solid and dashed lines are the linear regression fit and 95 % confidence interval for the regression slope, respectively. No smoothing was applied for the regression fit.

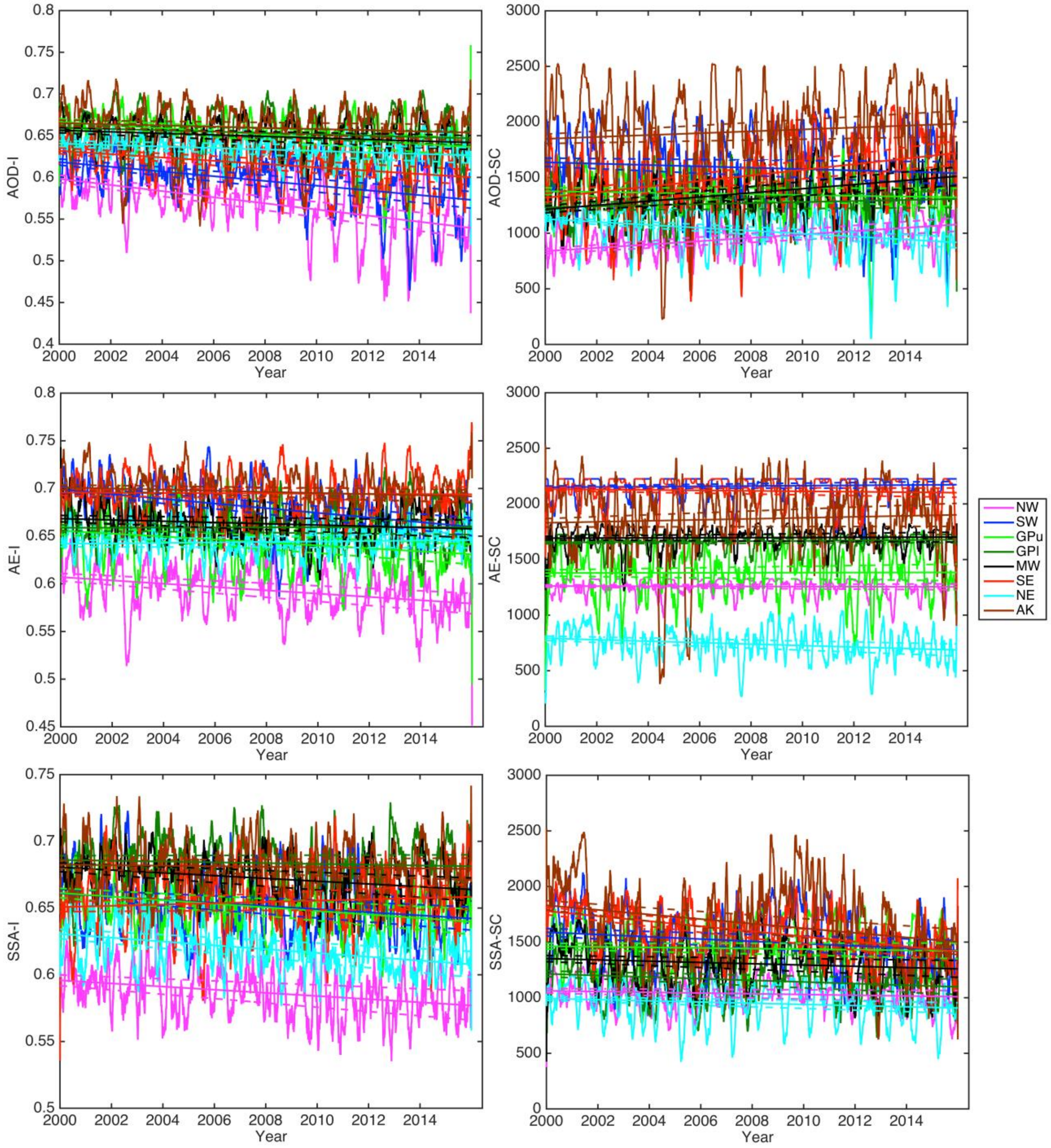

Figure S3. As in Figure S2 for trends in Moran's I (I) and scales of spatial coherence (SC) of AOD, AE, and SSA.
